# Analysis of the Prevalence of and Factors Associated with Hearing Loss in Korean Adolescents

**DOI:** 10.1371/journal.pone.0159981

**Published:** 2016-08-11

**Authors:** Seok Min Hong, Il-Seok Park, Yong Bok Kim, Seok Jin Hong, Byungho Lee

**Affiliations:** 1 Department of Otorhinolaryngology-Head and Neck Surgery, Dongtan Sacred Heart Hospital, Hallym University College of Medicine, Hwaseong, Korea; 2 Department of Internal medicine, Yes Hospital, Yongin, Korea; Universita degli Studi di Perugia, ITALY

## Abstract

**Background:**

Hearing loss can lead to a number of disabilities, subsequently reducing the quality of life. In general, hearing thresholds of adolescents are better than adults and the elderly. However, occasionally, adolescents acquire hearing loss for a number of reasons. In this study, our goal was to estimate the prevalence of hearing loss in the Korean population and to investigate the factors related to hearing thresholds in adolescents.

**Methods:**

A cross-sectional study was conducted using data from the Korean National Health and Nutrition Examination Survey (KNHANES) between 2010 and 2012. We enrolled a total of 1,658 participants, ages 13 to 18 years. We investigated the prevalence of hearing loss and the factors associated with hearing thresholds at various frequencies (0.5, 1, 2, 3, 4, and 6 KHz).

**Results:**

Weighted prevalence of unilateral and bilateral hearing loss in Korean adolescents was 2.2% and 0.4%, respectively. Weighted prevalence of hearing thresholds ≥ 20 dB at speech and high frequencies were 3.1% and 5.0%, respectively, for unilateral hearing loss and 0.7% and 1.9%, respectively, for bilateral. Age group, tympanometric data, and household income were significantly related to unilateral or bilateral hearing thresholds ≥ 20 dB at speech frequencies. Earphone use in noisy places was associated with bilateral hearing thresholds ≥ 20 dB at high frequencies.

**Conclusions:**

The prevalence of hearing loss in Korean adolescents was 2.6% using the general standard threshold associated with hearing loss. However, the prevalence of hearing thresholds ≥ 20 dB for speech and high frequencies was much higher. The results from this study provide an estimate of hearing loss in adolescents and suggest the need for early detection and hearing preservation programs among adolescents.

## Introduction

Hearing loss is currently one of the most prevalent, chronic conditions. Recent estimates by the World Health Organization report that 360 million individuals, 5.3% of the global population, suffer from permanent disabling hearing loss [1]. There are several causes of hearing loss, including genetic predisposition, congenital deafness, aging, infectious diseases (e.g., meningitis, chronic ear infections), ototoxic drugs, and exposure to excessive noise. Of these factors, aging is a well-known risk factor and age-related hearing loss begins in the third decade of life, initially involving sounds at high frequencies [2–4]. However, we often observe hearing loss in adolescents. Some studies have reported an increased risk associated with increasing exposure to leisure noise from listening to personal music players in young adults ≤ 18 years [5,6]. That said, other studies have found no link between the level of leisure noise and hearing loss [7, 8].

Independent of the cause of hearing loss, minimal and unilateral permanent damage may result in poorer educational test performance, higher incidence of failed grades, and greater dysfunction in areas such as behavior and self-esteem [9, 10]. Early detection of hearing loss provides the opportunity to restore and prevent these negative side effects. Despite the concerns associated with hearing loss, few studies have used an audiometric test to understand the role of demographics and the associated risk factors for hearing loss in adolescents on a nationwide scale [11].

In this study, we aimed to determine the prevalence of hearing loss in South Korean adolescents and to analyze the risk factors associated with hearing loss using data from the 2010–2012 Korean National Health and Nutrition Examination Survey (KNHANES).

## Materials and Methods

The institutional review board at the Korean Centers for Disease Control and Prevention (KCDC) approved the protocol used in this study and obtained written consent from the parents, next of kin, or guardians on behalf of the adolescents enrolled in our study.

### Study population

This study is based on data from the 2010–2012 KNHANES, a cross-sectional and nationally representative survey conducted by the KCDC. KNHANES has been conducted periodically since 1998 to assess the health and nutritional status of the non-institutionalized population of Korea. Participants were selected using a proportional, allocation systematic sampling with multi-stage stratification.

In total, 1,658 patients (886 male, 772 female) ages 13–18 years (junior and high school students) were enrolled in this study. Applying the recommended weighted values from the KNHANES, the total effective frequencies analyzed were 4,047,854 (2,187,468 male, 1,860,386 female).

### Survey

Participant demographics, including age group (junior 13–15 years of age, or senior [high-school students 16–18 years of age]), sex, residence type (apartment or non-apartment), town (urban or rural area), household monthly income ($2,000 or less, $2,000–4,000, and more than $4,000), and noise exposure history were categorized as personal risk factors. Noise exposure history was surveyed in detail and was classified into the following four categories: 1) earphone use in noisy places; 2) occupational noise, which includes at least 3 mo of work in an excessively noisy environment (e.g., manufacturing);, 3) non-occupational noise, including exposure to loud noise (e.g., vehicles and loud music) for >5 h per week; and, 4) momentary noise, which included exposure to brief loud sounds (e.g., gunfire and explosions). In addition, we sought tympanometric findings likely to affect the hearing threshold (otitis media or drum perforation). Abnormal tympanometry was defined as (1) no peak; (2) a peak pressure below minus 100 daPa; or, (3) a peak pressure above plus 100 daPa.

An audiometric examination was administered to all participants. Air-conduction pure-tone thresholds were obtained in a soundproof booth using an automatic audiometer (GSI SA-203; Entomed Diagnostics AB, Lena Nodin, Sweden). Supra-auricular headphones were used and the otolaryngologist provided basic instructions to the participants regarding the automated hearing test. Automated testing was programmed according to a modified Hughson-Westlake procedure using a single pure tone for 1–2 sec. The lowest level at which the subject responded to 50% of the pure tone was set as the subject’s threshold. The automated hearing test had good reliability and validity, comparable to the manual pure-tone audio test [12, 13]. Participants’ hearing thresholds were evaluated by allowing the subject to push a button when they heard a tone-like sound, similar to the method for measuring conventional pure-tone audiometry. The following frequencies were tested: 0.5, 1, 2, 3, 4, and 6 KHz. Results were recorded immediately.

We investigated hearing loss prevalence using the following criteria. Hearing loss was defined as a pure tone average (PTA) of the following frequencies: 0.5, 1, 2, and 3 KHz at a threshold of ≥ 26 dB HL. Moderate to profound hearing loss was defined as a pure-tone hearing threshold level of ≥ 41 dB. For early detection of hearing loss in adolescents, we investigated the prevalence of adolescents with hearing thresholds ≥ 20 dB HL in speech (average of thresholds at 0.5, 1, 2, and 3 KHz) and high frequencies (average of thresholds at 3, 4, and 6 KHz). Lastly, we investigated the factors associated with hearing thresholds ≥ 20 dB HL.

### Data analysis

KNHANES participants were not randomly sampled. The survey was designed using a complex, stratified, multistage probability-sampling model. To obtain representative prevalence information from the data, it was necessary to consider the power of each participant as representative of the Korean population (sample weight). Survey sample weights were calculated by considering the sampling rate, response rate, and age/sex proportion of the reference population.

We performed cross-tabulation analysis using a complex sampling method. Two-tailed analyses were conducted using SPSS (ver. 17.0; SPSS Inc., Chicago, IL, USA), and a *P*-value < 0.05 indicated statistical significance.

## Results

Of the 1,658 participants, 1,534 (weighted value: 3,770,012) completed a bilateral audiometric examination. Overall, the weighted prevalence of unilateral and bilateral hearing loss among Korean adolescents was 2.2% (confidence interval [CI]:1.3–3.7, n = 82,673/3,770,012) and 0.4% (CI:0.2–0.9, n = 15,652/3,770,012), respectively. The prevalence of unilateral and bilateral moderate to profound hearing loss was 1.0% (CI: 0.5–1.7, n = 36,115/3,770,012) and 0.3% (CI: 0.1–0.9,12,417/3,770,012), respectively (Table 1).

**Table 1:** Estimated prevalence of hearing loss in South Korean adolescents.

	Unweighted prevalence,%	Weighted prevalence, %
	(n = 1,534)	(n = 3,770,012)
		[95% CI]
Hearing loss		
Unilateral	2.3 (36)	2.2 (82,673)
		[1.3–3.7]
Bilateral	0.5 (7)	0.4 (15,652)
		[0.2–0.9]
Moderate-to-profound hearing loss		
Unilateral	1.3 (20)	1.0 (36,115)
		[0.5–1.7]
Bilateral	0.3 (4)	0.3 (12,417)
		[0.1–0.9]

Hearing loss: the averages of thresholds > 25 dB at frequencies of 0.5, 1, 2, and 3 kHz; moderate-to-profound hearing loss: the averages of thresholds > 40 dB at frequencies of 0.5, 1, 2, and 3 kHz, CI: confidence interval.

Weighted prevalence of adolescents with unilateral and bilateral hearing thresholds ≥ 20 dB at speech frequencies was 3.1% (CI: 2.1–4.5, n = 116,995/3,770,012) and 0.7% (CI: 0.4–1.3, n = 26,627/3,770,012), respectively. The prevalence of unilateral and bilateral hearing thresholds ≥ 20 dB at high frequencies was 5.0% (CI: 3.8–6.7, n = 190,268/3,770,012) and 1.9% (CI: 1.2–3.1, n = 73,031/3,770,012), respectively (Table 2).

**Table 2:** Estimated prevalence of adolescents with hearing thresholds ≥ 20 dB.

	Speech frequency		High frequency	
	Unweighted Prevalence,%	Weighted Prevalence,%	Unweighted Prevalence,%	Weighted Prevalence,%
	(n = 1,534)	(n = 3,770,012)	(n = 1,534)	(n = 3,770,012)
		[95% CI]		[95% CI]
Unilateral	3.1(48)	3.1 (116,995)	5.2 (80)	5.0 (190,268)
		[2.1–4.5]		[3.8–6.7]
Bilateral	0.8(13)	0.7 (26,627)	1.7 (26)	1.9 (73,031)
		[0.4–1.3]		[1.2–3.1]

Speech frequency: average hearing thresholds at frequencies of 0.5, 1, 2, and 3 kHz; high frequency: average thresholds at frequencies of 3, 4, and 6 kHz, CI: confidence interval

Cross tabulation analysis by complex sampling of participants with hearing thresholds ≥ 20 dB at speech frequencies indicated that high-school age (16–18 years) and abnormal tympanometric results were associated with a unilateral hearing threshold ≥ 20 dB (*P* = 0.018, *P* = 0.044, respectively). Furthermore, a household income of $2,000 or less was associated with a bilateral hearing threshold ≥ 20 dB (*P =* 0.011) (Table 3).

**Table 3:** Cross-tabulation analysis by complex sampling of unilateral and bilateral hearing thresholds ≥ 20 dB at speech frequencies.

	Unilateral		Bilateral	
	*χ2*	*P*	*χ2*	*P*
Age group (high-school)	5.079	0.018 ^a^	0.366	0.538
Sex	0.444	0.621	1.127	0.306
Resident in town	0.428	0.578	2.539	0.158
Residential type	0.863	0.412	0.240	0.561
Low household income	2.667	0.303	7.625	0.011 ^a^
Earphone use in noisy places	4.005	0.106	0.190	0.680
Occupational noise	0.841	0.361	0.076	0.810
Non-occupational noise	3.715	0.218	0.335	0.592
Momentary noise	0.036	0.861	0.033	0.781
Abnormal tympanometry	3.609	0.044 ^a^	0.073	0.803

Speech frequency: average hearing thresholds at frequencies of 0.5, 1, 2, and 3 kHz, CI: confidence interval

^a^ *P* < 0.05

For participants with hearing thresholds ≥ 20 dB at high frequencies, no factor was associated with a unilateral hearing threshold ≥ 20 dB. Only earphone use in noisy places was associated with a bilateral hearing threshold ≥ 20 dB (Table 4).

**Table 4:** Cross-tabulation analysis by complex sampling for unilateral and bilateral hearing thresholds ≥ 20 dB at high frequencies.

	Unilateral		Bilateral	
	*χ2*	*P*	*χ2*	*P*
Age group	0.040	0.860	1.711	0.181
Sex	1.341	0.377	1.866	0.281
Resident in town	0.006	0.951	0.981	0.426
Residential type	0.949	0.385	1.849	0.188
Household income	3.402	0.238	0.590	0.788
Earphone use in noisy places	0.022	0.901	4.510	0.027 ^a^
Occupational noise	0.177	0.675	0.223	0.712
Non-occupational noise	1.144	0.274	4.809	0.193
Momentary noise	0.002	0.976	0.694	0.416
Abnormal tympanometry	2.661	0.081	0.211	0.736

High frequency: average of hearing thresholds at 3, 4, and 6 kHz,

^a^ *P* < 0.05

## Discussion

In this study, we demonstrated that the weighted prevalence of hearing loss in Korean adolescents is 2.6% (unilateral and bilateral). The prevalence of the Korean population ≥ 19 years is 20.5% [14]. Although the prevalence of adolescents with hearing loss in this study was less than the adult population, it should not be overlooked. Hearing loss in children and adults may result in limited social activity, reduced quality of life, or psychological problems (e.g., feelings of isolation and exclusion, depression, and cognitive disorders) [15]. Of the estimated 98,325 adolescents with hearing loss, 82,673 adolescents had unilateral hearing loss, whereas the majority of the adult and geriatric population had bilateral hearing loss. These findings suggest that hearing loss in adolescents may be due to causes other than physiologic changes, such as aging. Also, in the early phase of noise-induced hearing threshold shifts (NITS), only one ear was damaged when a single frequency was involved [16].

In general, the standard for conventional hearing loss is ≥ 26 dB at average threshold during pure-tone audiometry. However, normal hearing in children is defined as a hearing threshold > 20 dB [16]. Therefore, we investigated the prevalence of adolescents with hearing thresholds ≥ 20 dB HL for speech and high frequencies. In our present study, we found that the unilateral and bilateral prevalence of adolescents with hearing thresholds > 20 dB HL was 3.1% and 0.7%, respectively, which was much higher than that based on 26 dB at average threshold. The prevalence of hearing loss at high frequencies was nearly three times that based on 26 dB of pure tone average.

Previously, a nationally representative study was published reporting demographic and audiometric results for adolescents residing in the United States. In this study, they reported that the prevalence of any hearing loss increased significantly from 14.9% between 1988–1994 to 19.5% during 2005–2006 [17,18]. However, in this study, hearing loss was defined as an average of low-frequency or high-frequency pure tone thresholds over than 15 dB in either ear. And compared to our study, the rates of prevalence reported in this study were higher than the rates we observed. A different study suggested that pure tone audiometry methods need to be standardized and that identification of minimal hearing loss should be performed using careful evaluation in the large-scale study [19].

We also evaluated associations between demographic and other factors, and hearing thresholds ≥ 20 dB at speech and high frequencies. Aging is a well-known risk factor for hearing loss but we found that being of high-school age was associated with a unilateral hearing threshold ≥ 20 dB at speech frequencies. This was not attributable to aging; all subjects were young. Also, a relatively low household monthly income ($2,000 or less) was associated with a bilateral hearing threshold ≥ 20 dB at speech frequencies.

No type of noise exposure was associated with unilateral or bilateral hearing thresholds ≥ 20 dB at speech frequencies. Of the types of noise investigated, earphone use in noisy places was associated with a bilateral hearing threshold ≥ 20 dB at high frequencies. It has been reported that portable music players can have deleterious effects on hearing thresholds in adolescents [19]. Noise exposure is an important risk factor for hearing loss. Although such exposure may be involuntary, many individuals are exposed voluntarily to noise, including those who listen to music on personal music players [20]. Unlike speakers, earphones can be used in public places. Earphones are, therefore, preferred by most individuals. However, by blocking the external auditory canals, earphones can elevate the output power by 7–9 dB, as compared to headphones that cover the entire external ears. In addition, more users of earphones, as compared to users of speakers, tend to elevate the sound volume to cope with environmental noise [21]. Of the 11 adolescents that initially showed bilateral high-frequency hearing loss (the non-weighted data), nine adolescents had relatively elevated high-frequency hearing thresholds at low frequency (a relatively high-frequency hearing loss: notching evident at 3, 4, and 6 kHz; hearing loss at least 15dB below those of the higher thresholds at 500 or 1,000 Hz).

But, using of TDH-style earphone, calibrated in a IEC303 couple, collapsing ear canal, and the presence of ear wax might result in an elevation of high-frequency thresholds [22,23], and also, some young children under 5 years were reported to have the mild sensorineural hearing loss [24]. And, these factors should be considered when we face the results in population-based large scale study.

Occupational or non-occupational noise was not associated with a unilateral or bilateral hearing threshold ≥ 20 dB at speech and high frequencies. In fact, most adolescents of the present study were students, and very few were exposed to occupational noise (they did not work). Neither non-occupational noise nor momentary noise were associated with hearing loss.

In adolescents, any association between hearing thresholds and noise remains controversial. Dehnert et al. and Williams et al. failed to find any relationship between leisure noise and hearing thresholds [7, 8]. However, leisure noise was associated with hearing loss in other studies [25, 26].

Another report suggested that while some leisure-noise is hazardous level, the relationship between noise and hearing loss is yet to be determined [27]. All of the study designs, subjects, noise evaluation methods, and modes of analysis varied in previous reports. We suggest that our present study is a well-designed larger-scale work analyzing various types of noise exposure using National Health and Nutrition Examination Survey data; we employed complex sampling analysis using weighted numbers.

Tympanometry abnormalities are associated with unilateral hearing thresholds ≥ 20 dB at speech. Tympanometry abnormalities include ear drum perforation, otitis media, impacted cerumen, or eustachian tube dysfunction. All participants with tympanometry abnormalities have the possibility of acquiring conductive hearing loss. Hearing loss due to tympanometry abnormalities can sometimes be recovered, but hearing loss due to noise exposure is usually permanent. Moreover, adolescents with hearing loss can be asymptomatic, preventing early detection in these individuals. In particular, high frequency hearing loss is not noticed until it progresses to the point of affecting speech and communication. It is thought that for hearing preservation in adolescents, early detection of hearing loss is important and hearing tests using a standard hearing threshold ≥ 20 dB should be administered.

One limitation was that KNHANES did not obtain data at 8 KHz, thus we analyzed threshold data ≤ 6 KHz. Data at a higher frequency may provide more detailed information on hearing thresholds.

## Conclusions

This study estimated the prevalence of hearing loss in adolescents and the results suggest the need for early detection, as well as hearing preservation programs, among adolescents.
